# Neural Sensitivity to Odorants in Deprived and Normal Olfactory Bulbs

**DOI:** 10.1371/journal.pone.0060745

**Published:** 2013-04-08

**Authors:** Francisco B. Rodríguez, Ramón Huerta, Maria de la Luz Aylwin

**Affiliations:** 1 Grupo de Neurocomputación Biológica, Dpto. de Ingeniería Informática, Escuela Politécnica Superior, Universidad Autónoma de Madrid, Madrid, Spain; 2 BioCircuits Institute, University of California, San Diego, La Jolla, California, United States of America; 3 Programa de Fisiología y Biofísica, Facultad de Medicina, Universidad de Chile, Casilla, Santiago, Chile; The University of Plymouth, United Kingdom

## Abstract

Early olfactory deprivation in rodents is accompanied by an homeostatic regulation of the synaptic connectivity in the olfactory bulb (OB). However, its consequences in the neural sensitivity and discrimination have not been elucidated. We compared the odorant sensitivity and discrimination in early sensory deprived and normal OBs in anesthetized rats. We show that the deprived OB exhibits an increased sensitivity to different odorants when compared to the normal OB. Our results indicate that early olfactory stimulation enhances discriminability of the olfactory stimuli. We found that deprived olfactory bulbs adjusts the overall excitatory and inhibitory mitral cells (MCs) responses to odorants but the receptive fields become wider than in the normal olfactory bulbs. Taken together, these results suggest that an early natural sensory stimulation sharpens the receptor fields resulting in a larger discrimination capability. These results are consistent with previous evidence that a varied experience with odorants modulates the OB's synaptic connections and increases MCs selectivity.

## Introduction

Neuronal representations of sensory stimuli are shaped by sensory experience and the modification of these representations may underlie changes in perceptual abilities. The neuronal representations in vertebrates initiate with the activation of the olfactory receptor neurons (ORN) by odorants. The ORNs, expressing the same receptor molecule [1], project to two glomeruli in each OB [2]. Different odorants activate distinct, but partially overlapped, combinations of OB glomeruli. These spatial maps of activated glomeruli constitute the main odorant coding scheme in the OB [3], [4]. Within the OB there is a complex inhibitory network that transforms the spatial activation into spatio-temporal activity patterns [5]–[10]. The OB network of reciprocal and lateral connectivity between mitral cells (MCs) and granule cells [8], [11] is shaped by olfactory experience [12]. More specifically, early olfactory deprivation reduces the number of inhibitory neurons [13], [14], adjusts the pattern of inhibitory connectivity [15] and, slows the morphological development of mitral cells [16]. On the contrary, an enriched olfactory environment increases the number of inhibitory neurons [17]. Functionally, early olfactory deprivation increases the fraction of MCs activated by an odorant [18], [19] and slows the developmental changes in membrane conductance [16]. Furthermore, the integrity and plasticity of the inhibitory network is required to discriminate similar odorants [11] and improve novelty detection and sensitivity [20] in invertebrates. In agreement with the structural and functional changes, early sensory deprivation modifies odorant discrimination and identification [21] as well as the responsiveness of the MCs to olfactory stimulation [22]. Specifically, there is an increase in the fraction of MCs that exhibit odorant responses [18], [19], [23] and local field potentials in the OB [19], consistent with a decrease in the inhibitory input onto MCs. Behaviorally, a recent study showed an increased odorant discrimination of a binary mixture [24]. However, the effects of early olfactory deprivation in odorant discrimination and information storage in the OB, the first processing stage of the olfactory pathway, have not been elucidated.

In this study we examined the properties of the MC activity changes induced by early sensory deprivation in terms of neural sensitivity. Sensitivity is defined as the fraction of neurons that show positive responses (excitatory and inhibitory) to 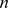 stimuli out of a total of 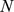. Lastly, we estimated the theoretical MCs stimuli discrimination and information storage capacities.

Our results show that despite the remarkable anatomical changes in the early deprived OB, MCs ongoing and odorant triggered activity is comparable in both the normal and deprived olfactory bulb. Specifically, in the absence of olfactory stimulation, the MCs firing rate is similar in deprived and normal OBs, consistent with the homeostatic hypothesis [25]. Odorant induced MC responses, excitatory and inhibitory, show similar variations of frequency around the baseline, indicating that the deprived OB adjusts the overall MC sensitivity to olfactory stimulation. Interestingly, the fraction of MCs that show odorant responses increases in the deprived OB, likely due to the lack of olfactory experience. In fact, MCs in the deprived OB respond to more odorants, indicating that they are less selective and carry less information about the odorant than MCs from an OB exposed to natural stimulation. These results suggest that the olfactory bulb adjusts the overall activity levels to the environmental stimuli as proposed by Cleland et al [26] and more interestingly, natural sensory stimulation sharpens the odorant representations of odorants.

## Materials and Methods

### Animal and Surgical Preparation

Surgical and experimental techniques described in detail in [19] were carried out in strict accordance with the recommendations in the Guide for the Care and Use of Laboratory Animals of the National Institutes of Health. The protocol was approved by the Committee on the Ethics of Animal Experiments of the University of Chile (protocol CBA-079). Surgery was performed under anesthesia, and all efforts were made to minimize suffering and distress. In brief, sprague-Dawley rat pups (PND1) were anesthetized with ice and their left nostril was permanently closed by swift cauterization. Pups remained with their mothers until the 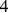
th week, then they were kept in separate cages with food and water ad libitum until the recording session. Animals were maintained in a reversed 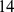-h light/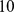-h dark cycle and all experiments were done in the dark phase of the cycle. Adult animals (P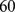 and P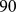 from 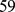 to 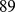 days of sensory deprivation) anesthetized with a mix of ketamine (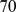 mg/kg), acepromazine (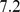 mg/kg) and atropine (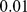 mg/kg), and the anesthesia level was maintained with urethane (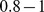 g/kg) i.p. supplemented, as necessary, to abolish any sign of distress. Temperature was maintained at 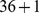
°C with an electrical blanket. Before the recordings, the deprived nostril was fully reopened using a small surgical cauterizer. Subsequently, the animals were positioned in a stereotaxic apparatus and the dorsal surface of both OBs were exposed. After the protocol was finished, the animals were euthanized with a barbiturate overdose.

### Recording Techniques

Unitary activity was recorded with a 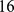-channel linear-probe (CNCT, Michigan, USA). Electrode impedances were between 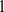 to 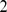 MOhms (1 kHz) and contact separation was 50 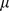
m. All penetrations were performed perpendicular to the OB surface and the electrode was lowered until MCs action potentials were observed in the center of the electrode array. In each animal, recordings were obtained from both deprived and non-deprived OBs by alternating penetrations at each side. The unitary activity was amplified (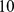
K), filtered 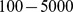 Hz, and digitized at 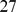 KHz, using custom designed PC software.

### Odorant Stimulation

**Figure 1:**
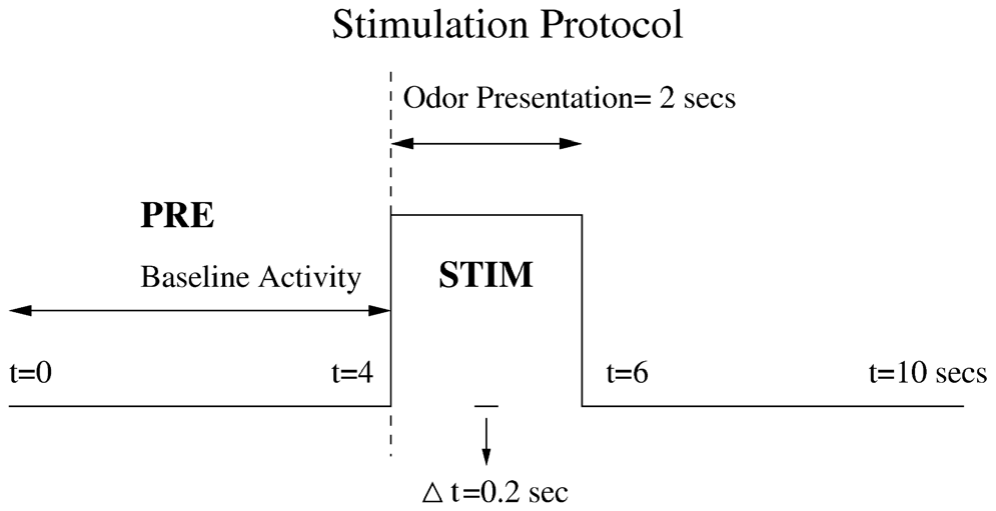
Trial and odorant stimulation protocol. Each trial (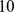 s) starts with 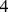 seconds of clean air named prestimulus (PRE) epoch, followed by the odorant stimulation epoch (STIM) starting at 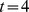 seconds. Four different stimuli were applied in sequence and this sequence was repeated 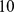 times: clean air or control, r-carvone, isoamylacetate and hexanal. The interstimulus time was 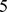 s.

Olfactory stimuli were presented with a custom made olfactometer by a PC controlled solenoid valves. Pressurized air, from commercially purified tanks, previously humidified was streamed to an empty tube or a tube with an odorant diluted in mineral oil (total volume 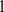 ml), whose output was connected to an inverted funnel facing the animal's nose. We used monomolecular odorants: r-carvone, isoamylacetate and hexanal (Sigma-Aldrich, cSt. Louis, MO) diluted in a 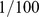 ratio. These odorants have previously been used in anesthetized rats to trigger glomeruli activity in the OB [3], [27]. As shown in fig Fig. 1, each trial consisted of 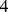 s of clean air (PRE), followed by a 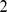 s odorant stimulus (STIM) and 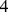 s of clean air. Trials were separated by 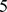 s of inter–trial interval. Odorant sequence was the following: air, r-carvone, isoamylacetate, and hexanal. To reduce odorant adaptation there was no repetition of the same odorant in consecutive trials.

### Spike Sorting Algorithm

**Figure 2:**
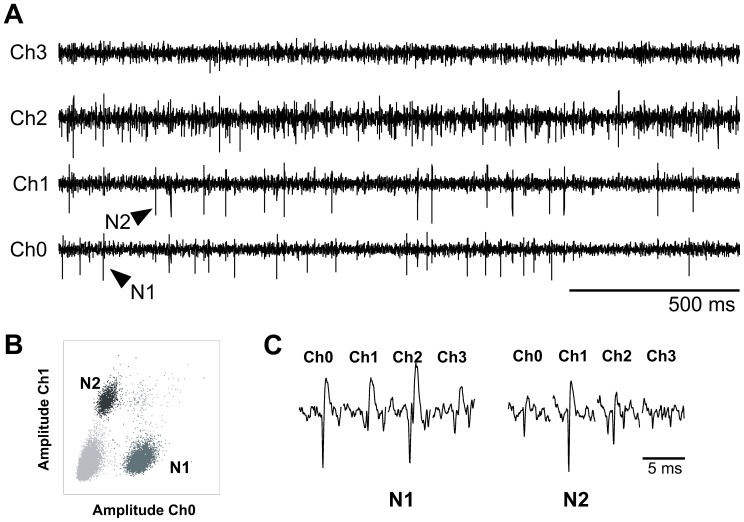
Example of signal recording and single-unit sorting in the normal OB. A: the top 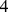 traces correspond to the filtered signal (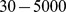 Hz) from 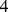 electrodes (channel 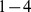). The black arrows indicate the spikes corresponding to the neuron 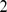 in channel 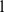 and neuron 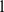 in channel 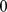. B: scatter plot of waveform peak-to-peak amplitudes recorded in channel 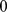 vs. channel 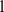. Two clusters clearly emerge, corresponding to the single-unit activity shown in A. C: An example of the spike waveforms of the 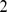 clusters shown in B.

For each data set, spike separation was performed by an interactive custom computer program [28]. An example of spike sorting is shown in Fig. 2. The spike parameters (spike amplitude, time to peak, principal component) for two out of the sixteen recording channels of the linear electrode were displayed in two dimensional scatter plots, revealing a clustering of the data points. Ellipses were drawn around distinctively clustered data points and the values corresponding to each cluster were assigned a unique color. The clusters can then be iteratively redefined in as many projections as needed to uniquely define a particular single unit. An example of the clustering resulting from the plot of peak-to-peak amplitudes recorded in neighboring channels are shown in Fig. 2 B. In this example, channel 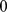 vs channel 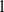 exhibits two clusters, corresponding to the spikes in Fig. 2 A. The spike waveforms of these cells are shown in Fig. 2 C. Once a unique cluster was defined, the spike train of each cell was computed by recovering the time stamp of each data point in the cluster. The extracted spike train for each cell was stored with a 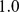 ms resolution. Whenever two clusters were not separated, the spikes were pulled together and classified as multiunit. The existence of a refractory period [29] in the firing rate histogram was used as additional criteria to classify the single units. This multiunit spikes were not analyzed in this manuscript.

### Detection of MC Responses to Odorants

The detection of MC's responses to odorant stimulation (see Fig. 1) can be difficult due to the small difference in firing rate between the PRE and STIM epochs [4], [19]. A neuron's response can be represented as a binary decision problem: a response to a stimulus occurs if there is a statistical difference in the firing rate between PRE and STIM epochs. Typically, the baseline discharge statistics is used as a reference to determine a response during the stimulation period. In addition, the firing rate can also vary largely due to the respiratory modulation [4], [29]. Consequently, stimulus-evoked changes in firing rate decrease significantly if we average over the complete stimulation epoch. In other words, the statistics of MC firing rate during stimulation does not differ considerably from baseline statistics. To improve the reliability of the MC response detection we used a methodology based on [30], that reduces the effect of the firing rate variability in the response detection based on the response probability, which is described in the next section.

### Probability of MC Response to Odorant Stimulation

To determine whether a particular MC responds to a stimulus should be ideally addressed by a maximum likelihood ratio defined as the quotient between the probability activity of observation when we know there is MC response and the probability activity of observation when we know there is not MC response [31]. Note that the Neyman-Pearson lemma [32] is very clear in this respect: the likelihood ratio test is the most powerful for a given significance level of a test, 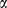. Unfortunately, it is not possible to obtain the probability of the observations given a response because a MC may or may not fire/respond to the specific stimulus. To circumvent this problem, we can calculate a tight bound of the response probability [30]. This method has the advantage that it does not require implicit assumptions about the underlying and unknown probability distribution. To estimate the response probability we used bootstrapping techniques [33], and a complete description of the method is given in [30]. To estimate the response probability, we first define a window to measure the conditional response to external stimulation (normally the time of the odor presentation). A specific MC can discharge 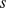 times in the time window of 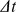 seconds where 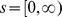. To discriminate if the MC activity is the result of odorant stimulation or noise variations, we denote the event 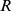 as the response to a stimulus, and the event 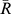 represents the absence of a response. Then we estimated the probability of having 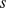 responses in the absence of stimulus for the total MC population. For a set of observations 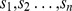 in 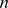 different odorant presentations, ideally, it would be convenient to calculate 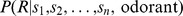 but this is not possible. However, a tight bound can be calculated through the complementary probability or negative response probability, 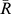, *i.e.*, 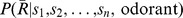 = 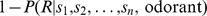.

By applying the Bayes' theorem to the conditional or posterior probability 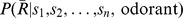 we can obtain.
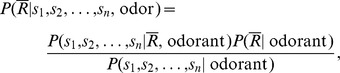
where 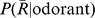 the “prior” probability for the non-response random variable. It is “prior” in the sense that it does not consider any information about the stimulus. The probability 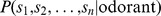 operates as a normalizing constant. The estimation of 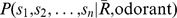 represents the probability distribution of a set of observations 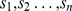 in 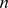 trials, where there is no response to the stimulus. We then calculate the probability distribution of this set of observations 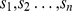 without any applied stimulus using the baseline data that can be expressed as 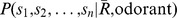 = 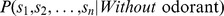 = 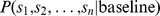.

The unknown “prior” probability 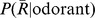 cannot be obtained by a straightforward calculation. However, we know that.
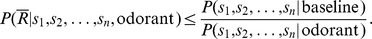



Finally, the complementary probability for no response is.
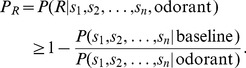
(1)


The right-hand side of this equation represents the lower bound of the response probability 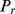 (see details in [30]).

To obtain the lower bound estimator for Eq. 1 we calculated the probability distributions 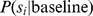 and 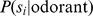. Using bootstrapping, we obtained the non-parametric distributions. The probability distribution 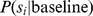 for each cell is calculated across trials during PRE epoch (see Fig. 1), corresponding to the time interval between 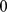 and 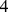 seconds, in successive 200 ms bins with steps of 100 ms. The probability distribution 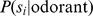 is calculated using the same window duration and step for each cell during the STIM epoch (see Fig. 1). These probability distributions are combined in Eq. 1 to obtain the MC response probability, 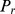. Since the underlying baseline activity is not always stationary due to the respiratory driven oscillatory discharge of the MCs [4], [29], we used an additional analysis to reduce the false positive responses. To perform this correction, we applied the same procedure to the trials with clean air and determined the values of 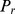 that had the lowest level of false positives. Moreover, because the stimulation epoch last 2 seconds, the response detection test should be positive during consecutive windows. On the contrary, in the absence of stimulation the probability of having two or more consecutive windows with false positives should be negligible. Thus, the 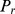 bound value should maximize the responses during the STIM epoch and minimize them during the PRE or baseline epoch (false positives). To estimate the 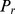 bound value, we calculated in the case of clean air or control (odorant 0) the percentage of detected responses (false positives) for increasing values of 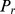.

**Figure 3:**
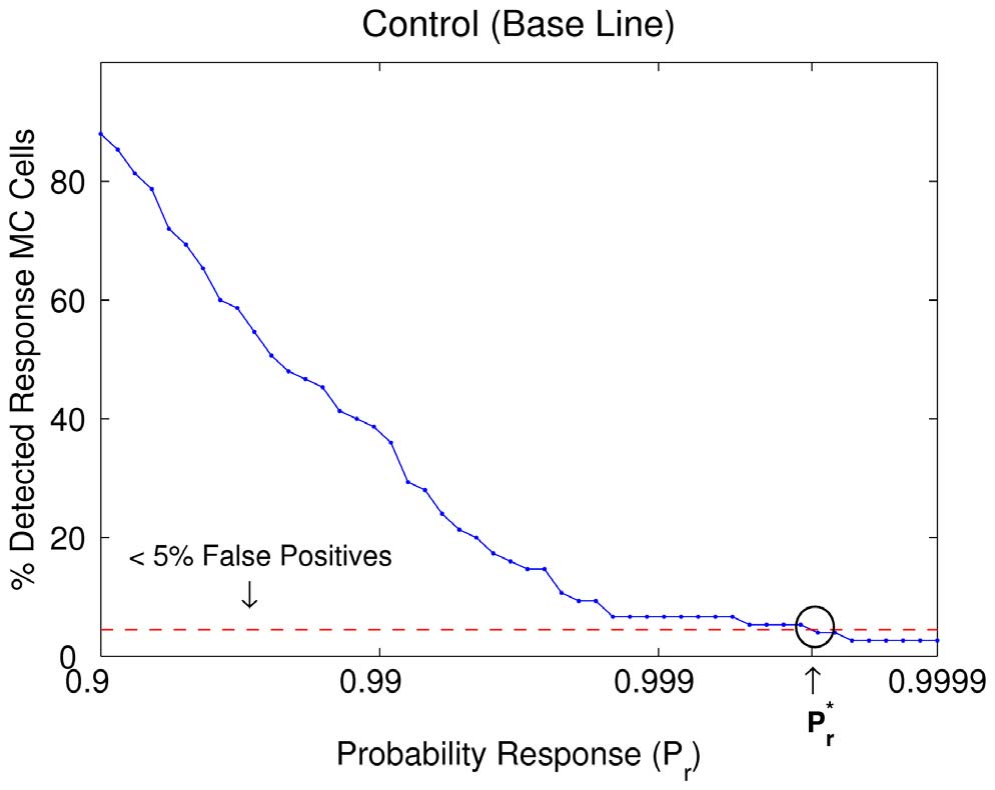
Percentage of responses estimated by the probability method as a function of the *P_r_* in the absence of odorant stimulation. The percentage of detected responses, calculated for all MCs including all the trials with clean air, decreased significantly as the 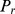 value is increased. This criteria was used to decide which value of the probability was selected for a desired maximum of false positive. We choose a maximum of false positive 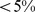 (see dash line) corresponding to a value 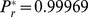.

The percentage of detected responses in MCs as a function of the 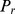 in the absence of odorant stimulation is illustrated in Fig. 3. As expected, in the presence of clean air, there is a progressive decrease in the percentage of detected responses as 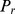 value increases, reducing the percentage of false positives up to 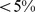 for values of response probability greater than 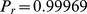. Based on this, we selected this specific bound probability as the response criterion 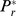, see dashed line in Fig. 3.

## Results

From 10 animals we recorded from a total 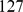 MCs, 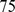 cells were unequivocally classified as single units and selected for further analysis. Of these, 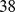 MCs from 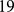 sites were recorded in the deprived and reopened OBs and 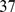 MCs from 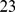 sites in the normal OBs. During the experiments, animals were exposed to 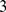 different monomolecular odorants and clean air, all presented to the nose in urethane anesthetized rodents. Each stimulus was presented for 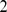 seconds interleaved with the other 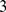 stimuli. The stimulus set was repeated 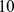 times with the same stimulus presented every 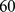 seconds (see Fig. 1) to reduce odorant adaptation.

### MC's Activity in Deprived vs. Normal OB

**Figure 4:**
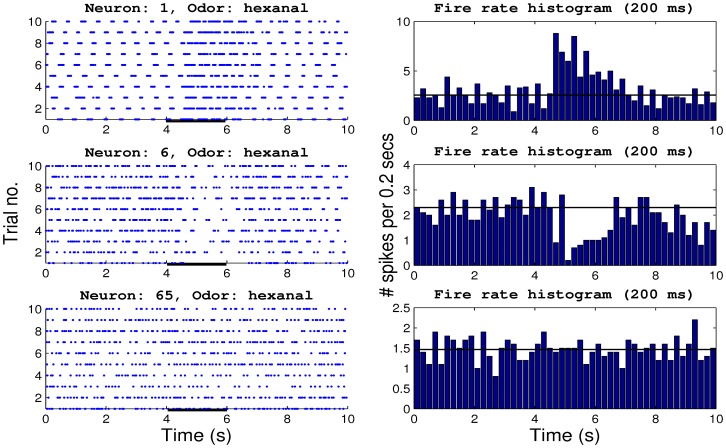
Examples of three different types of MCs responses to odorant stimulation. The left panels show the spike rasters for three different cells during odorant stimulation. The right panels show the firing rate histograms calculated in 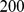 ms bins for the same cells. The continuous line represents the mean firing rate during the baseline epoch. The MC on the top shows an excitatory response, the middle MC shows an inhibitory response and the bottom cell does not respond to odorants.

The MCs firing rates exhibited different properties depending on the cell identity and olfactory stimuli. An example of MCs response types in the presence of hexanal are shown in Fig. 4. In this example, the spike rasters of three MCs illustrate a modulation of the firing rate during odorant stimulation in the top and middle cell (left panels Fig. 4). The corresponding firing rate histograms (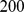 ms bin) are shown in the right panels of Fig. 4. In this figure, the horizontal solid line represents the mean firing rate during the baseline epoch. The cell shown on top exhibits an excitatory response in the presence of hexanal. On the contrary, the middle cell exhibits a robust inhibitory response and the cell at the bottom exhibits no response to hexanal.

**Figure 5:**
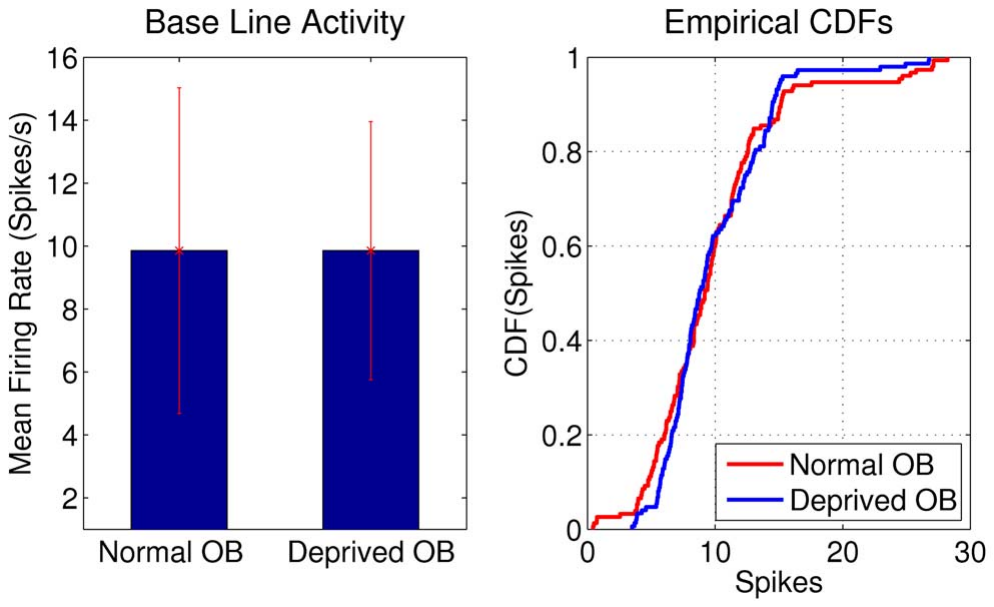
Mean firing rate of MCs in the normal and deprived OB during the baseline epoch. Mean firing rates were not significantly different in normal and deprived OBs (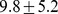 and 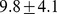, respectively, 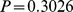 K-S test). We show cumulative distribution function of spikes in the normal and deprived OB for visual comparison. The variance (or SD) appears to be smaller in deprived OBs and we perform a K-S test for differences of the SD giving a 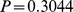.

Because early sensory deprivation reduces the number of granule cells [34], [35] and increases the excitability of granule inhibitory cells [15], it is reasonable to assume that there is a trade–off between the reduced inhibition due to the lower number of granule cells and the increase on the excitability of the remaining granule cells. In consequence, the MC's discharge in the absence of odorant stimulation in the deprived OB should remain within the range observed in the normal OB. To evaluate the consequences of inhibitory changes in the MC discharge in the absence of odorant stimulation, we evaluated the mean firing rate during baseline conditions. As expected, we found that the mean firing rate of the MCs in the deprived OB was not significantly different from the one observed in the normal OB (Fig. 5). Mean firing rate were 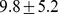 Hz and 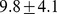 Hz, in normal and deprived OBs, respectively (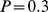 K-S test). This result is consistent with an homeostatic regulation of the baseline MC discharge in the deprived OB.

### Neural Sensitivity

**Figure 6:**
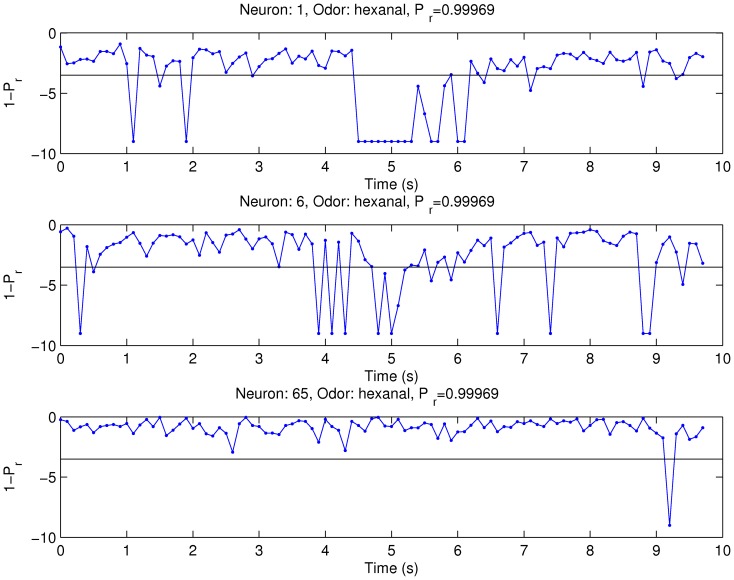
Examples of MC's odorant responses estimated with the probability method for the same cells shown in Fig. 4. The graphs represent the response probability in a 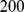 ms moving window in steps of 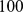 ms in a single trial. The response probability is shown in a logarithmic scale. The detection criteria (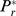) is indicated by the continuous line.

We went on to examine the consequences of olfactory deprivation, and the underlying inhibitory circuitry changes, in the odorant induced responses. In particular, we examined if MCs were more responsive to odorants in normal vs. deprived OB. A measure of MC’s responses to odorants in deprived vs. normal OB is the sensitivity defined as the distribution of neurons that respond to 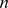 out of 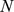 stimuli. We compared the sensitivity of the MCs to the 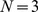 odorants in normal and deprived OBs. With the response probability method we estimated the response of each cell-odorant pair. An example of the response probability throughout the trial is shown in Fig. 6 for the same cells shown in Fig. 4. The ordinate (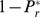) is shown in a logarithmic scale to facilitate the visualization of the responses that reach the 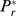 criterion. In this example, the maximal reliability in the lower bound estimator, 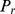, occurs on the stimulation epoch in the cell shown at the top graph, the middle graph shows an inhibitory response. With the same value of 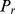, the cell in the bottom graph does not respond to the odorant.

**Figure 7:**
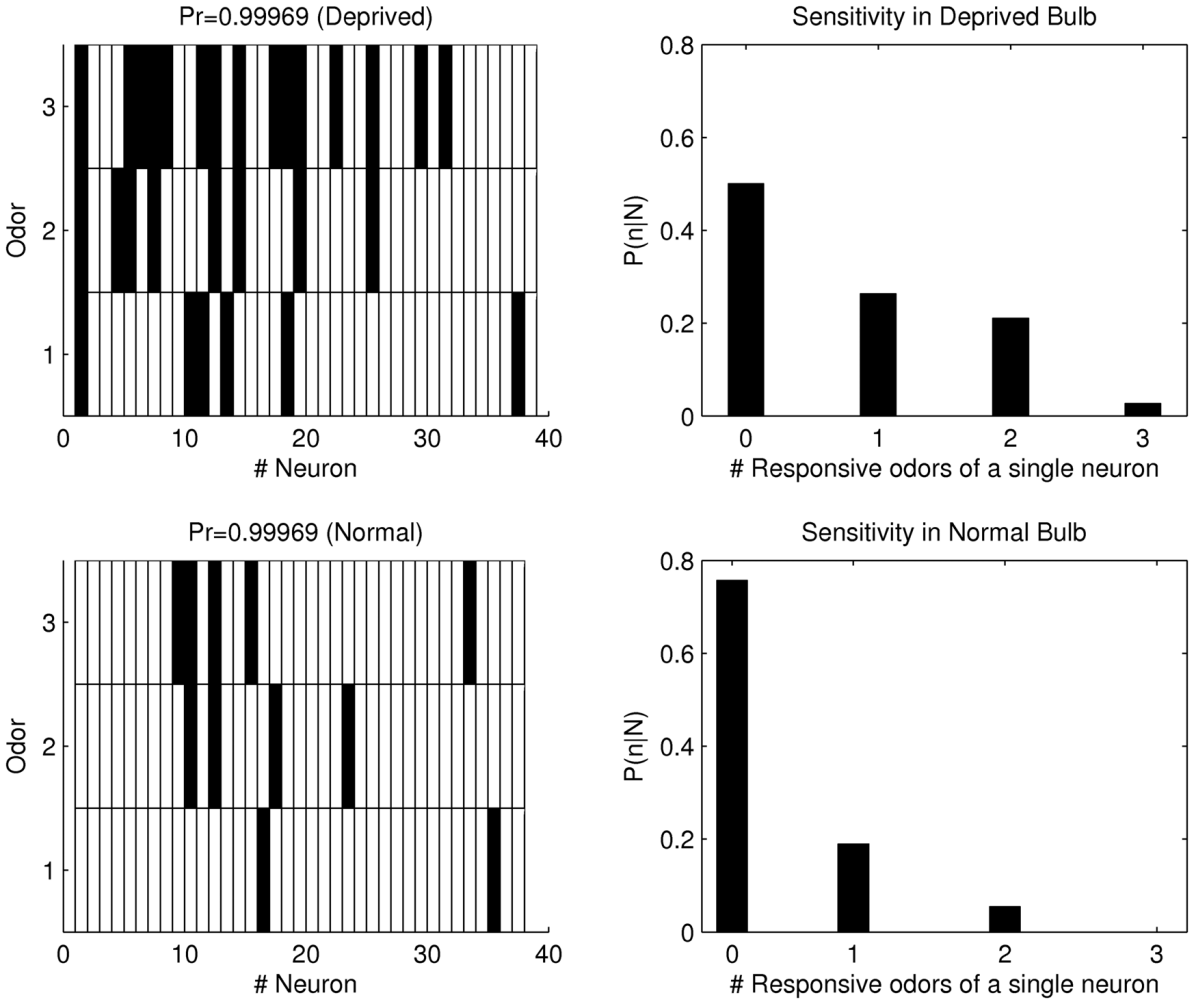
MCs responses to odorants in normal and deprived OBs. We used the lower bound estimation of probability response when 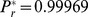. The left panels show the distribution of odorant responses (filled rectangles) for MCs. The numbers 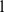, 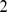 or 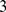 of the odorants correspond to r-carvone, isoamylacetate and hexanal respectively. The right panel shows the sensitivity of odorants for the normal and deprived OBs.

The distribution of responses for all MCs with the three stimuli is shown in Fig. 7. Each rectangle represents a cell-odorant combination and the odorant responses are indicated by filled rectangles. Note that MCs can respond with an excitation or inhibition, both events considered as neural responses. We found that 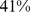 of MCs in the deprived OB respond to odorants compared to 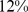 of the cells in the normal OB. This result indicates that the deprived OB has an increased sensitivity to odorant stimulation compared to the normal OB in anesthetized rats. To further describe the sensitivity of the MC population to the stimuli set, we calculated the distribution of neurons that respond to 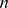 stimuli out of a total of 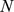 as shown in the right panels (Fig. 7). In the normal OB, the majority of MCs do not exhibit odorant responses. On the contrary, in the deprived OB there is a substantially higher fraction of responsive MCs. In normal OB, none of the cells responded to all three odorants and the fraction of cells that responded to 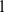 and 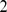 odorants was small. These results are quantified as the sensitivity values being 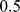 with no response, 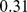 with one odorant, 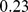 with two odorants and 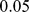 with three odorants in the deprived OB. In the normal OB, the sensitivity was 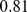 with no response, 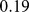 with one odorant and 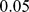 with two odorants. In summary, in the deprived OB the majority of the MCs respond to one or two odorants, while in the normal OB the majority of the MC respond to one odorant. Thus, the deprived OB exhibits an increased sensitivity to different odorants when compared to the normal OB.

### Intensity of the Odorant Responses

**Figure 8:**
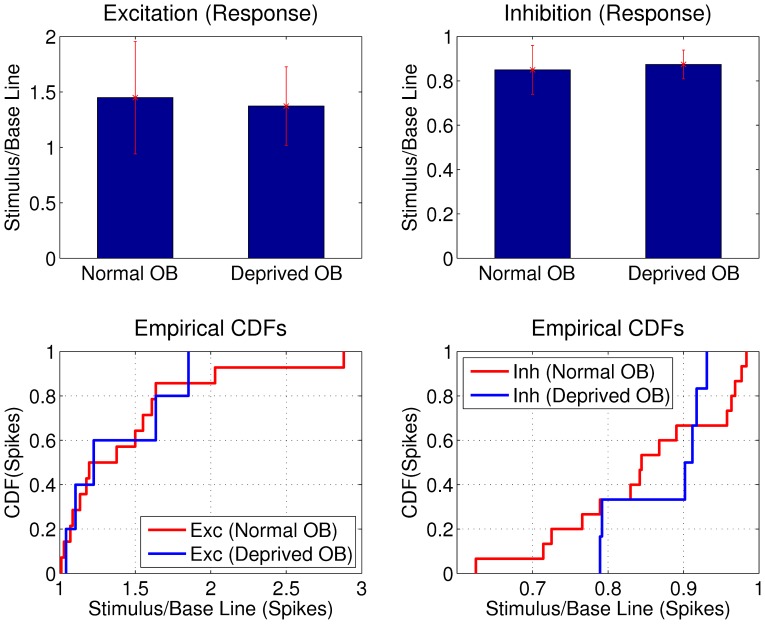
Mean firing rate ratio between stimulus and baseline epochs for the cells that exhibited an excitatory or inhibitory responses in normal and deprived OB. Ratios for excitatory (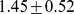 and 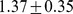) and inhibitory (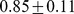 and 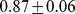) were not significantly different between normal and deprived OB (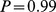 and 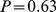 respectively, K-S test). We show for visual comparison, cumulative distribution function of spikes for the cells that exhibited an excitatory (Exc) or inhibitory (Inh) responses in the normal and deprived OB.

Because the reduction in the number of inhibitory granule cells in the deprived OB, the difference in the sensitivity to odorants observed between normal and deprived OBs could arise from differences in the intensity of the response evoked by odorants. If the odorants induce a stronger modulation of the firing rate in the deprived OB, the sensitivity of the method will detect more responses in the deprived OB. To determine if the responses from deprived and normal OBs had different intensity, we calculated the ratio of firing rate between stimulus and baseline epochs for each cells that exhibited excitatory or inhibitory responses. We found the mean ratio between stimulus and baseline epochs was not significantly different between normal and deprived OBs for excitatory and inhibitory responses. Specifically, the ratio for the excitatory responses increased about a 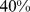 (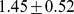 in normal and 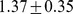 in deprived OB) and the ratio for inhibitory responses decreased around 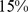 (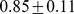 in normal and 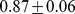 in deprived OB). As shown, in Fig. 8, these values are not significantly different between normal and deprived OB (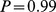 and 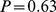 respectively, K-S test). In summary, the excitatory and inhibitory responses exhibited similar firing rate modulation in agreement with an homeostatic regulation of the total excitation and inhibition in the deprived OB during odorant stimulation.

**Figure 9:**
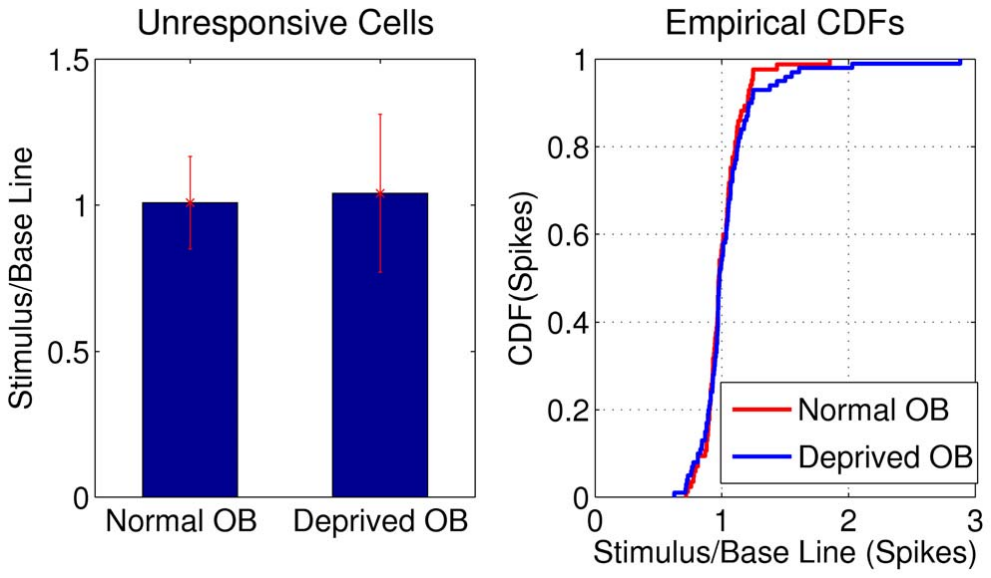
Mean firing rate ratio between stimulus and baseline epochs for the unresponsive cells in normal and deprived OB. Ratios for normal OB (1.01±0.16) and deprived OB (1.04±0.27) were not significantly different (*P* = 0.99, K-S test). We show cumulative distribution function of spikes in the normal and deprived OB for visual comparison.

Additionally, we corroborated if the firing rate ratio (stimulus/baseline epochs) for the MCs that did not exhibited odorant responses were different in the deprived and normal OB. As expected, the mean ratios were not significantly different in both conditions (see Fig. 9). These results indicate unresponsive MCs do not significantly modulate its mean firing rate during odorant stimulation.

Taken together, these results indicate that early sensory deprivation likely induces an homeostatic adjustment of the level of excitatory and inhibitory sensory induced activation in the OB. Notwithstanding, there is an increase sensitivity to different odorants in the deprived OB.

### Stimuli Potential Capacity vs. Response Overlap

Odorants activate a distributed combination of glomeruli representing a spatial code [36]. To examine the coding capacity of the OB given by the maximal number of different patterns constrained by the observed properties of MCs activation in the OB, we performed a qualitative analysis of the maximal capacity and compared it with the overlap of MCs activation. To improve the discrimination ability of different odorants one needs to distinguish the neural activities induced by these odorants. Since, the discrimination between odorants are dependent on the degree of collision between different induced activities, we estimate their overlap probabilities.

**Figure 10:**
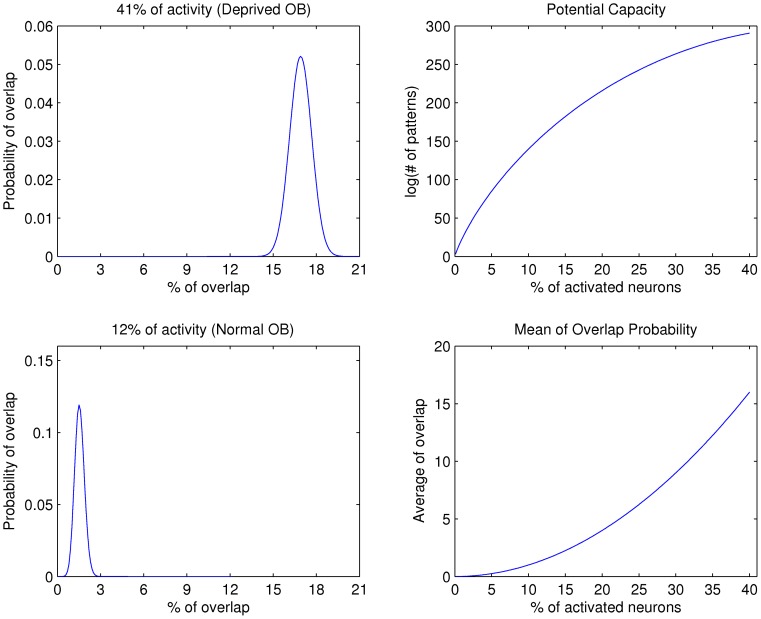
Theoretical estimation of odorant capacity vs. discriminability. The left panels show the probability of overlap calculated for the deprived and normal OBs that exhibits 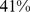 and 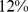 of responses, respectively. The right panels present the potential storage capacity and the mean overlap probability as a function of the percentage of activated neurons.

We define a set of binary numbers 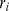, representing the MC responses, where the index 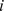 runs from 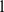 to 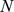. The numbers indicate whether a given neuron is activated (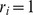) or not (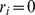) by the odorant. Assuming there is a vector of 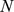 neurons of which 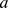 are activated; the maximal capacity of responses is given by the combinatorial number 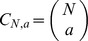. From this equation, a total of 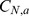 different patterns of 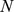 locations (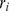 different binary number with 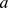 ones) that code each of 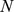-vector stimulus. The variation of the overlap between the different patterns of responsive neurons is related to the ability of the system to discriminate between different activity patterns. If there is more overlap between the activity patterns, the discriminability decreases. To examine the degree of discriminability for our data we estimated the probability of overlap between two random patterns of 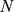 neurons with 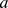 activated neurons (two 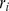 binary number with 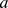 ones each of one). These calculations are described in [37], [38] and the probability is given by:
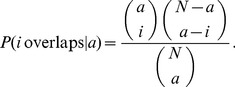
(2)


which represents the probability of having 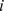 output collisions given a specific activation degree for the output system, 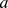. We only assume codes with a precise and specific level of activity (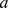 responsive neurons out of 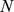 total neurons) will be present on the network. In consequence the probability distribution of the neural activity is centered in some particular level so not all the codes are equally probable or perhaps possible. We need to estimate the probability of overlapping at a particular level of activity. The next step would consist of compounding the conditional probability 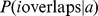 with the prior probability 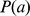: i.e. 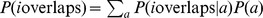. However, to estimate this “prior” probability we need to test different odorant concentrations in our experiments. Consequently we would obtain a representative sample of different activity levels in order to calculate this probability, 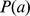. For our particular estimations we take the value of OB level activity in our experiments. We need to estimate the overlapping by assuming a particular level of activity, given the above equation. In Fig. 10 we show the values obtained from this equation. Left panels depict the probability of overlap for a system with 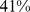 of activity, as the ones obtained in the analysis of deprived OB data, in contrast to a system with 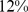 of activity as for the normal OB data. These results indicate the percentage of overlap is significantly reduced in a system with low levels of activation, i.e. 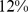 of activity as for the normal OB data. The mean of overlap probability for the normal OB is 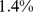, whereas in the deprived OB the mean value for overlap probability is 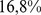. From this data, we can estimate the mean of overlap probability for different percentages of responsive neurons (both excitatory and inhibitory). As shown in Fig. 10, the average overlap probability increases with percentage of responsive neurons indicating that the odorant discrimination is more reliable when there is low activity patterns (low number of responsive cells). However, there is a trade-off between the ability to discriminate different odorants and the potential to store of different odorants. We can define the storage capacity for different patterns of 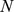 locations, as the combinatorial number 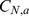 for a given level of activity 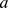 (see calculation above). This potential capacity was calculated for different percentages of activation in the OB. As shown in Fig. 10, when the potential capacity is increased, the average of overlap in the activity patterns increase in the same way and therefore the ability to discriminate different odorants is decreased.

## Discussion

The main objective of this work was to compare the properties of the MCs discharge from deprived and normal OBs in anesthetized rodents and estimate, from the theoretical standpoint, the discriminability and storage capacity of deprived and normal OBs. Our results show that the deprived OB maintains the basal level of activity in the absence of odorant stimulation, in agreement with homeostatic mechanisms that keep the system within a sensitive range to external stimulation. Homeostatic mechanisms for activity dependent excitability and synaptic strength regulation have been previously described in invertebrates as a result of action potential blockade [39], [40]. In the OB, early sensory deprivation reduces the number of granule cells and their synaptic density, causing an overall reduction of the inhibitory connectivity within the OB. There is also an increase in the excitability of granule cells [15], that appears to compensate for the reduction in inhibitory connectivity. In our recordings, the absence of changes of the spontaneous MC firing rate is consistent with an overall compensation of the inhibitory input onto MCs during baseline odorant free condition. To the best of our knowledge, this is the first report about the properties of the ongoing MCs activity in the deprived OB. Interestingly, our results are consistent with the homeostatic regulation of the OB circuitry that adjust the level of baseline activity to different levels of external drive [41].

In the presence of olfactory stimulation we found an increase in the incidence of excitatory and inhibitory responses in MCs from deprived OB when compared to the normal OB, indicating regulation of the activity levels during odorant stimulation. In summary, these results demonstrate an overall increase in the sensitivity of the deprived OB to olfactory stimuli. Despite the regulation of the overall OB activity levels during baseline and odorant stimulation, the deprived OB MCs activation patters are consistent with a reduced discrimination ability. In other words, the number of neurons involved in stimulus coding is larger in the deprived OB when compared to the normal OB. This reduction in the sensitivity of MCs is due to an increase in the excitatory as well as the inhibitory responses.

The adjustment of the overall OB activity levels during baseline and odorant stimulation is apparently inconsistent with a reduction in the inhibitory input onto MCs [15], as we assumed that a reduced inhibitory drive would increase the fraction of responses and the excitatory responses of the OB. Surprisingly, we found no evidence of an increase in the fraction of excitatory responses in the deprived OB, suggesting that there is an adjustment of the inhibitory input onto MCs during sensory deprivation. An alternative explanation for this inconsistency may arise from the fact that the inhibitory input onto MCs is conveyed by a lower number of inhibitory neurons, which may in turn decrease the diversity of the MCs responses, particularly those connected to the same glomeruli [10].

Other studies about the effect of early sensory deprivation on the olfactory pathway have shown an increase in the epithelial response to odorants [42], [43], which are predominantly excitatory in rodents [44]. An increase in the excitatory responses in the epithelia should increase the excitatory drive of MCs through glomerular synapses which should increase the number of MCs responding to odorants with an excitatory response. Again, our results are consistent with a regulation of excitatory as well as inhibitory MCs odorant evoked responses.

The consequences of the differences in the sensitivity of deprived and normal MCs can be explained in terms of stimuli discrimination, where the normal OB has a clear advantage in this sense. In a system with low activation levels, like the normal OB, the percentage of overlap is significantly reduced as shown in Fig. 10. The theoretical estimation of this reduction in the percentage of overlap indicates an increase in the discrimination of different combinatorial MCs activation.

As described in the last section, the OB needs to balance between the ability to discriminate different odorants and the potential to store different odorants, i.e. storage capacity. We show that there is a negative relation between discrimination and storage capacity, the higher the system discrimination the lower the system storage capacity (Fig. 10). A system with high discrimination could improve the storage capacity if we consider the time dimension in the neural code. Several studies indicate that the olfactory system uses spatio-temporal patterns of neural activation to encode odorants. This coding strategy has been examined in insects [11], [45], [46] and vertebrates [4], [47]–[49]. Therefore, if we consider a spatio-temporal coding, it can be theoretically demonstrated [50], that the maximal system storage capacity is reached when there is a minimal number of activated neurons for a given time. An additional advantage of a small degree of activation in the normal OB is that the neural system could use less time to process the odorant induced activity pattern, and of course less energy consumption. Fonollosa et al. [51] analyzed a spatial map of OB activation which does not consider the temporal information and it was obtained in dead animals previously exposed to a single odorant. Nevertheless, the low receptor correlation described by Fonollosa et al. [51] reflects the combinatorial activation of glomeruli by different odorants and its variations in the degree of activation, an additional dimension to the odorant activation of the OB. Therefore, it is possible to generate a network with a high activation but a low overlap, but it requires the maximization of mutual information between the inputs and outputs sets. Interestingly, the real problem arises when we consider the biological restriction of these networks, particularly if we compare the effect of the inhibitory network on the odorant responses and coding capacity to the same odorants.

The olfactory system detects, discriminate and identifies hundreds of different odorants which could be a single molecule type or a combination of several compounds. Our study aimed to compare the functional responses of MCs in normal and deprived OBs. The low number of odorants and the use of anesthetized animals are limitations of this study. We used a low number of odorants because the time necessary to test a higher number of odorants would substantially reduce the number of sites recorded for each animal, and increase the number of animals required. Furthermore, the use of anesthetized animals in this study minimized the firing rate variability due to the animals active modulation of the respiratory cycle. It is well known that the respiratory cycle is highly modulated in awake rodents by several factors such as novelty, previous learning, stimulus meaning such as appetite or aversive, etc. In our recordings, there was a constant respiratory rate reducing the variation of the firing rate due to the respiratory rate (see Fig. 4).

In summary, we compared the ongoing and odorant induced MCs activity in the normal and deprived OBs from the same animal. We have shown that the deprived OB retains a basal level of activity suggesting an homeostatic mechanism to keep the system in a sensitive range to external stimulation. Furthermore, the deprived MCs increase their excitatory and inhibitory responses when compared to the normal MCs during odor stimulation. We show an overall increase in the sensitivity of the deprived OB to olfactory stimuli versus normal OB. This means, that the number of neurons involved in stimulus coding is larger in the deprived OB when compared to the normal OB. Finally, we show from the theoretical standpoint, that in a system with low activation levels (normal OB), the percentage of overlap is significantly reduced, increasing the discrimination between activity patterns induced by different odorants.
